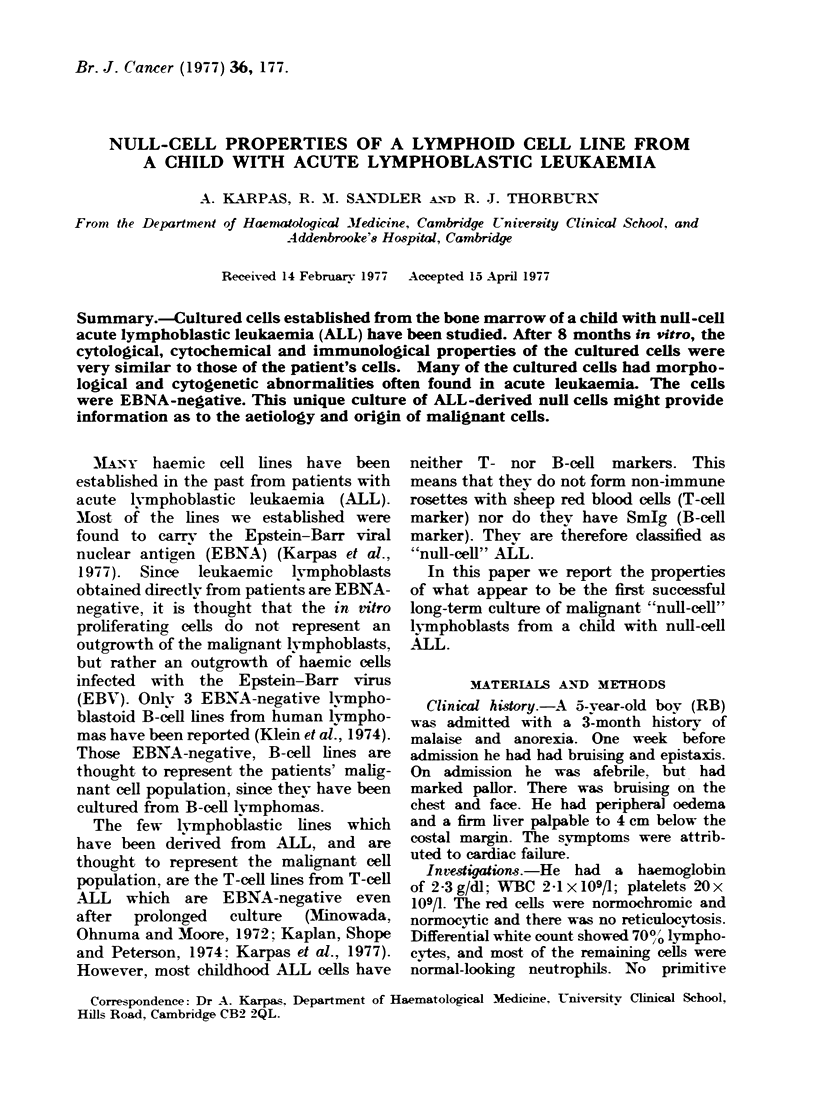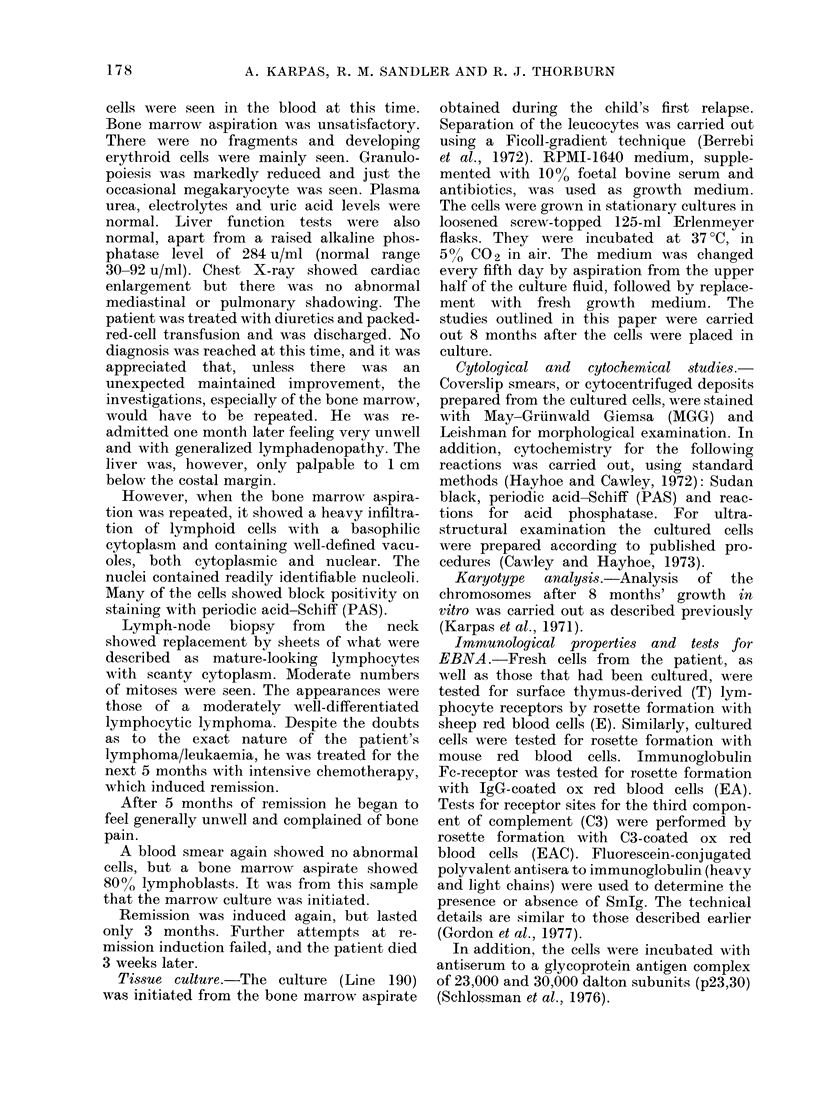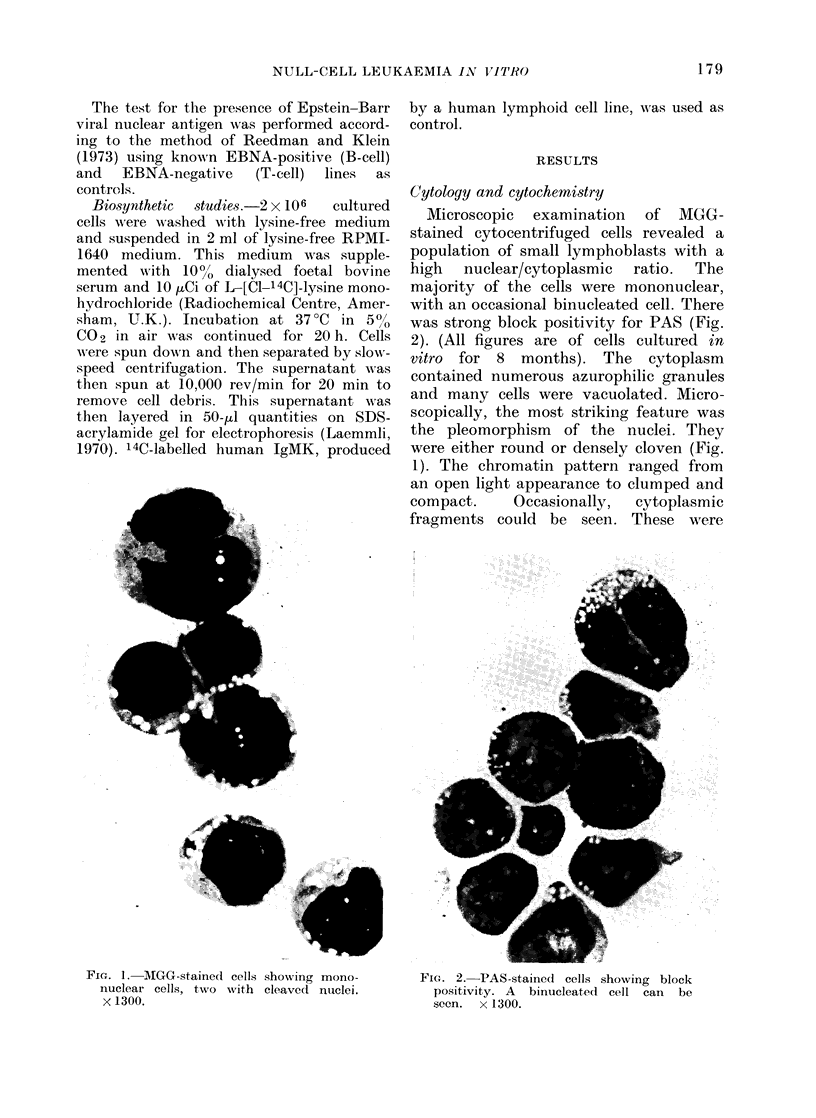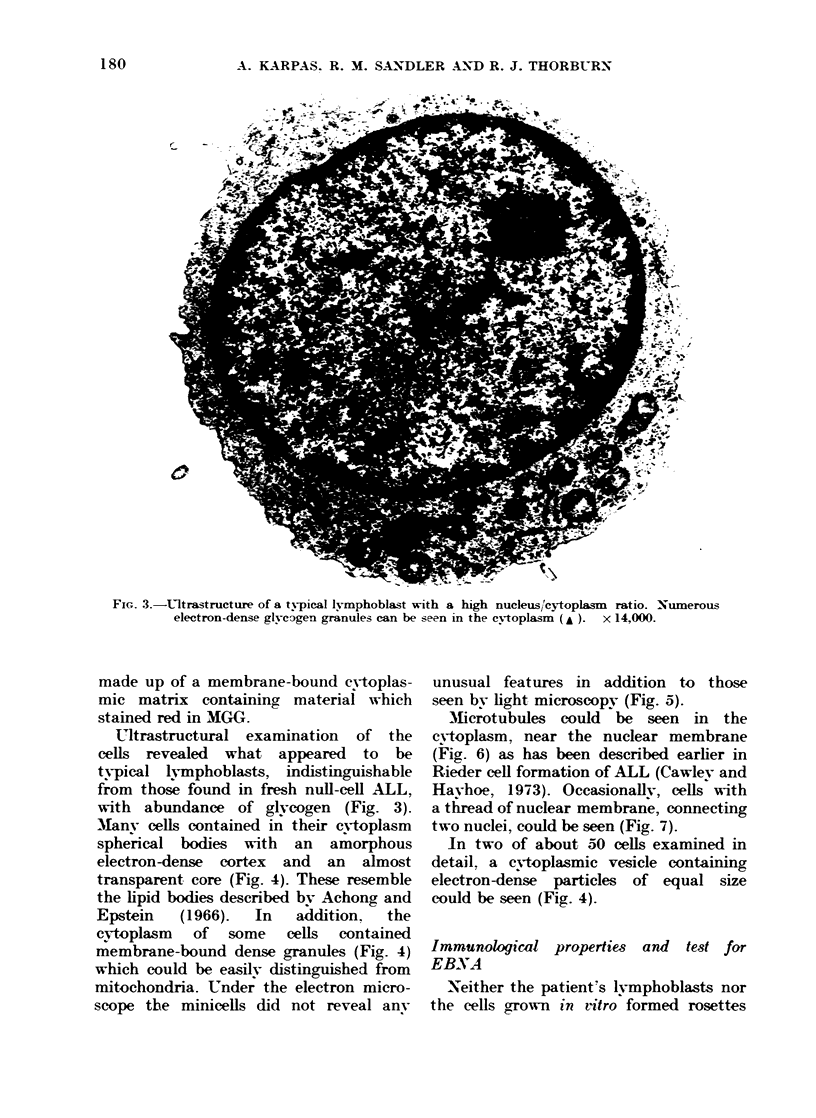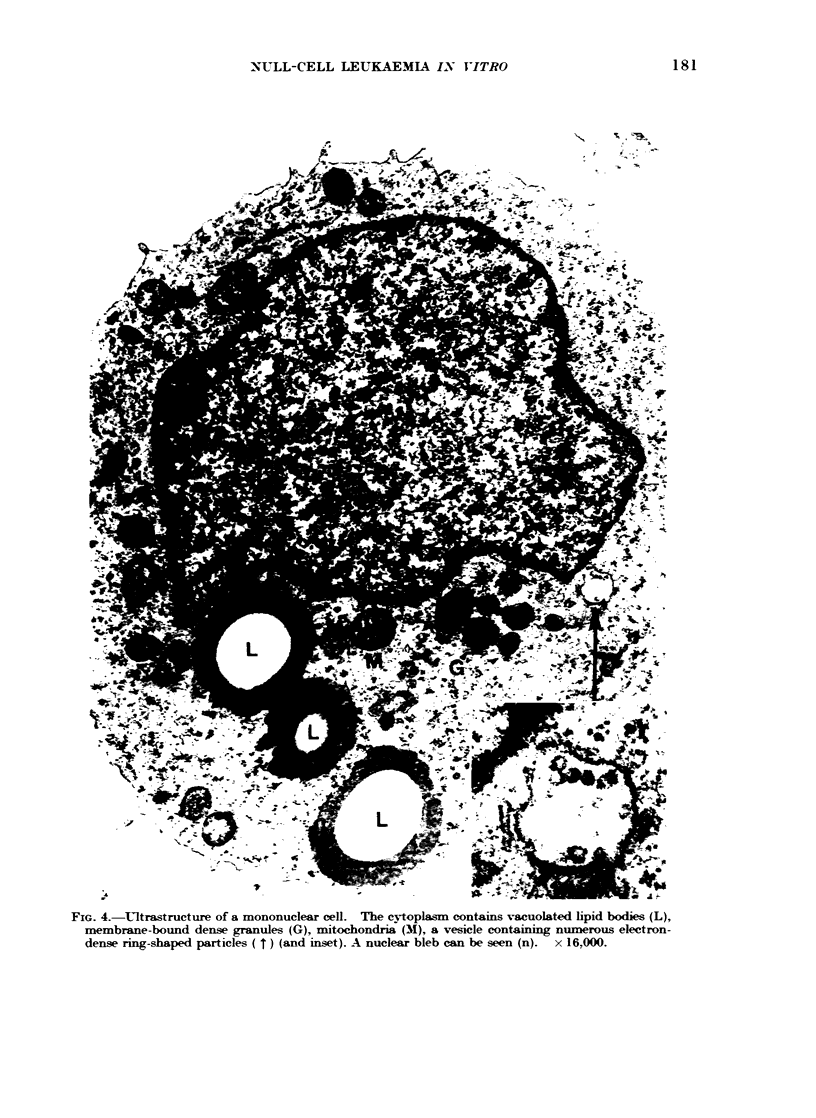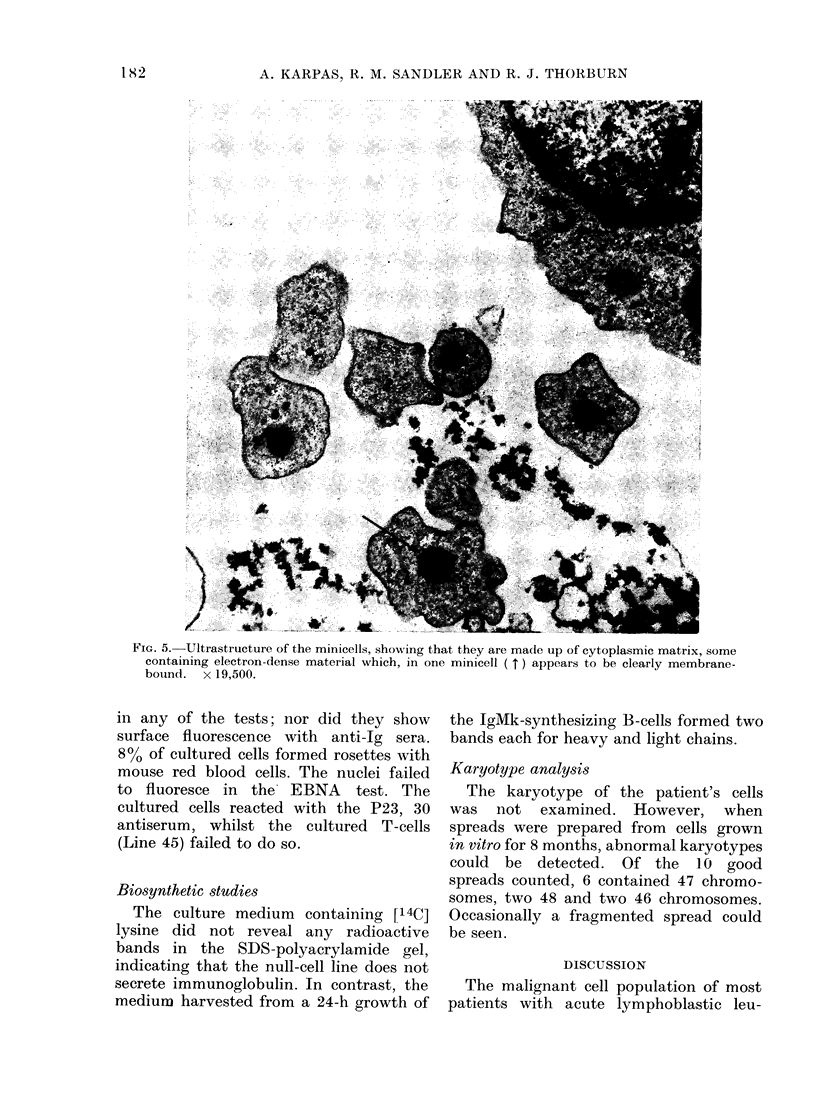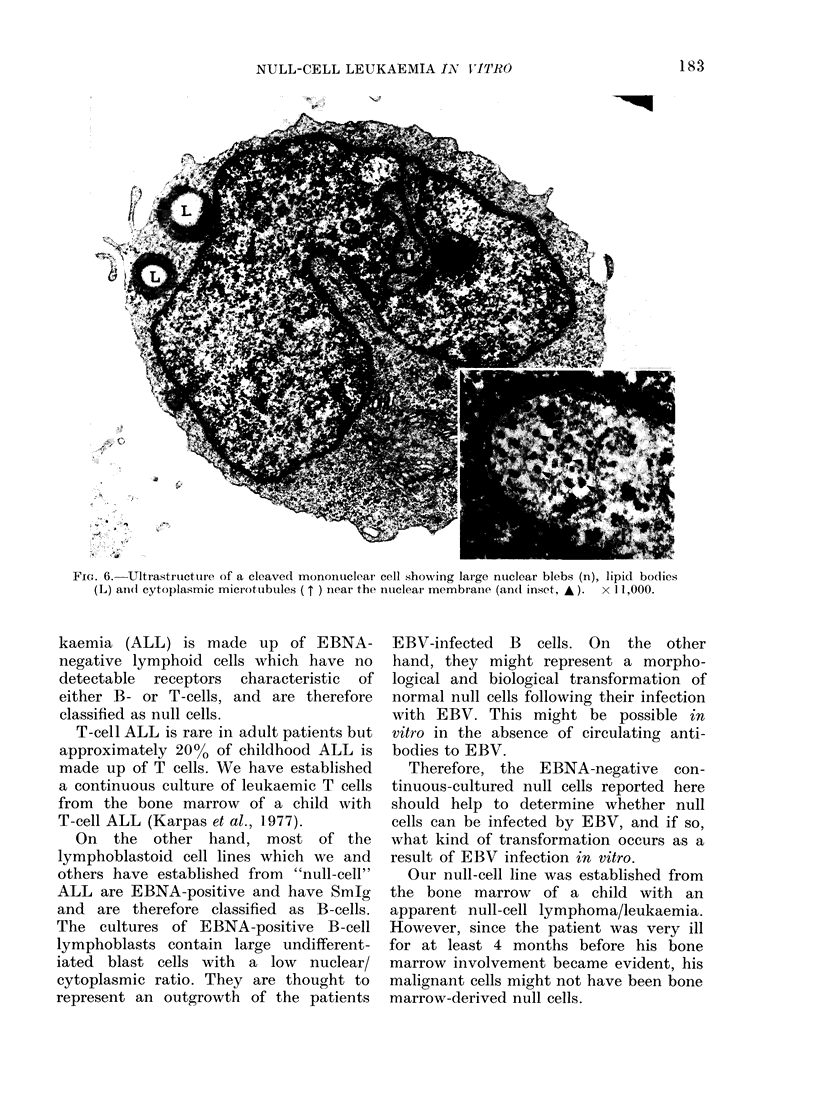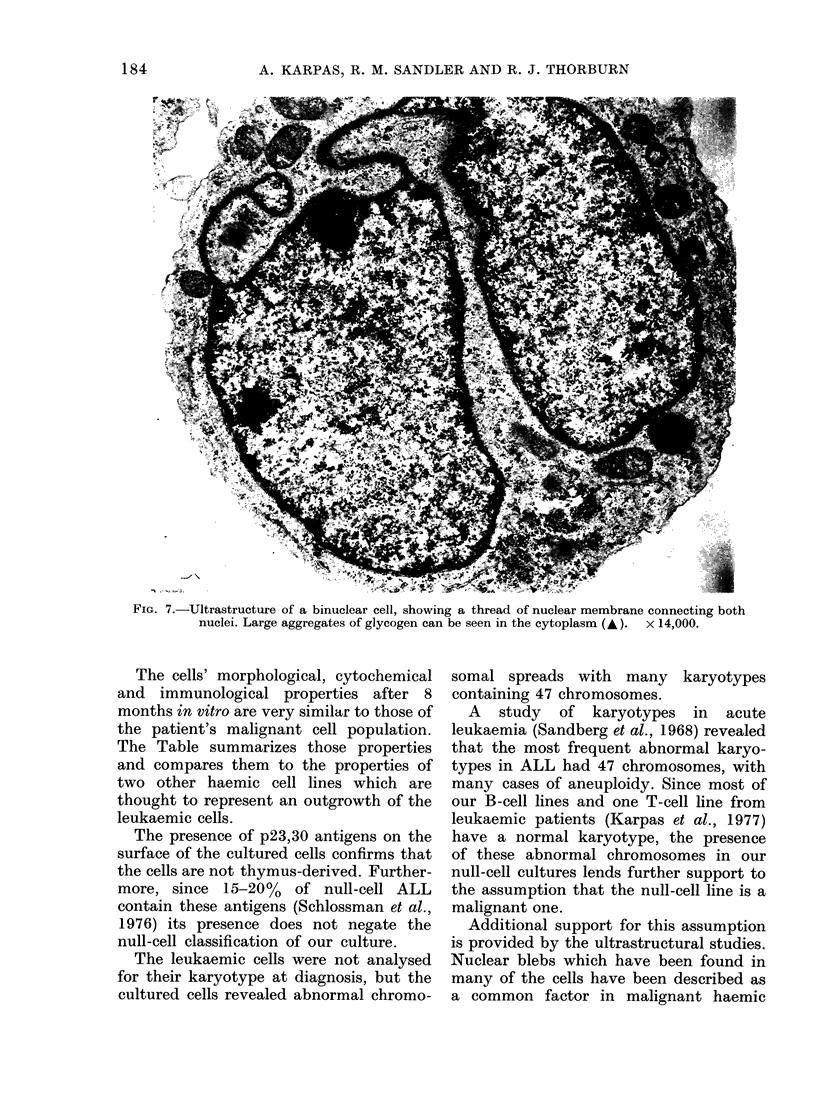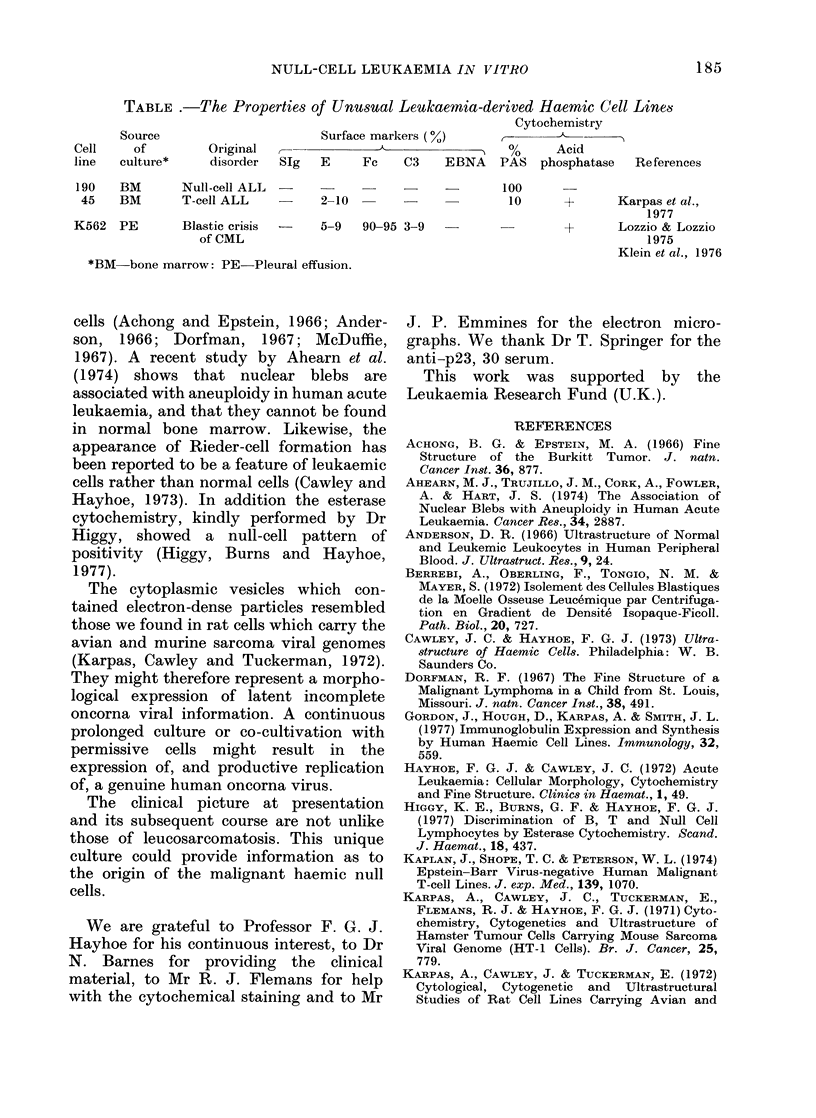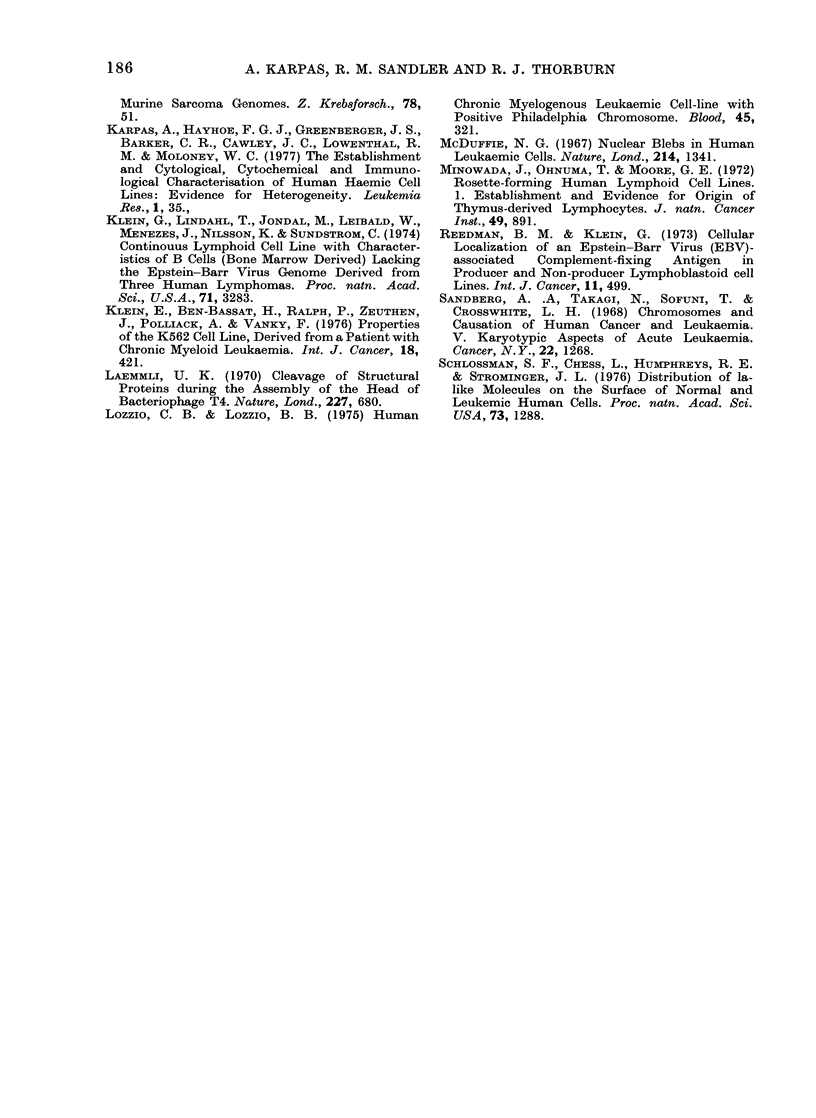# Null-cell properties of a lymphoid cell line from a child with acute lymphoblastic leukaemia.

**DOI:** 10.1038/bjc.1977.175

**Published:** 1977-08

**Authors:** A. Karpas, R. M. Sandler, R. J. Thorburn

## Abstract

**Images:**


Br. J. Cancer (1977) 36, 177.

### NULL-CELL PROPERTIES OF A LYMPHOID CELL LINE FROM A CHILD WITH ACUTE LYMPHOBLASTIC LEUKAEMIA

A. KARPAS, R. M. SANDLER AND R. J. THORBURN

*From the Department of Haematological Medicine, Cambridge University Clinical School, and Addenbrooke's Hospital, Cambridge*

Received 14 February 1977 Accepted 15 April 1977

**Summary.—Cultured cells established from the bone marrow of a child with null-cell acute lymphoblastic leukaemia (ALL) have been studied. After 8 months *in vitro*, the cytological, cytochemical and immunological properties of the cultured cells were very similar to those of the patient's cells. Many of the cultured cells had morphological and cytogenetic abnormalities often found in acute leukaemia. The cells were EBNA-negative. This unique culture of ALL-derived null cells might provide information as to the aetiology and origin of malignant cells.**

MANY haemic cell lines have been established in the past from patients with acute lymphoblastic leukaemia (ALL). Most of the lines we established were found to carry the Epstein–Barr viral nuclear antigen (EBNA) (Karpas *et al.*, 1977). Since leukaemic lymphoblasts obtained directly from patients are EBNA-negative, it is thought that the *in vitro* proliferating cells do not represent an outgrowth of the malignant lymphoblasts, but rather an outgrowth of haemic cells infected with the Epstein–Barr virus (EBV). Only 3 EBNA-negative lymphoblastoid B-cell lines from human lymphomas have been reported (Klein *et al.*, 1974). Those EBNA-negative, B-cell lines are thought to represent the patients' malignant cell population, since they have been cultured from B-cell lymphomas.

The few lymphoblastic lines which have been derived from ALL, and are thought to represent the malignant cell population, are the T-cell lines from T-cell ALL which are EBNA-negative even after prolonged culture (Minowada, Ohnuma and Moore, 1972; Kaplan, Shope and Peterson, 1974; Karpas *et al.*, 1977). However, most childhood ALL cells have neither T- nor B-cell markers. This means that they do not form non-immune rosettes with sheep red blood cells (T-cell marker) nor do they have SmIg (B-cell marker). They are therefore classified as "null-cell" ALL.

In this paper we report the properties of what appear to be the first successful long-term culture of malignant "null-cell" lymphoblasts from a child with null-cell ALL.

### MATERIALS AND METHODS

*Clinical history.*—A 5-year-old boy (RB) was admitted with a 3-month history of malaise and anorexia. One week before admission he had had bruising and epistaxis. On admission he was afebrile, but had marked pallor. There was bruising on the chest and face. He had peripheral oedema and a firm liver palpable to 4 cm below the costal margin. The symptoms were attributed to cardiac failure.

*Investigations.*—He had a haemoglobin of 2·3 g/dl; WBC $2{\cdot}1 \times 10^9$/l; platelets $20 \times 10^9$/l. The red cells were normochromic and normocytic and there was no reticulocytosis. Differential white count showed 70% lymphocytes, and most of the remaining cells were normal-looking neutrophils. No primitive

Correspondence: Dr A. Karpas, Department of Haematological Medicine, University Clinical School, Hills Road, Cambridge CB2 2QL.

cells were seen in the blood at this time. Bone marrow aspiration was unsatisfactory. There were no fragments and developing erythroid cells were mainly seen. Granulopoiesis was markedly reduced and just the occasional megakaryocyte was seen. Plasma urea, electrolytes and uric acid levels were normal. Liver function tests were also normal, apart from a raised alkaline phosphatase level of 284 u/ml (normal range 30–92 u/ml). Chest X-ray showed cardiac enlargement but there was no abnormal mediastinal or pulmonary shadowing. The patient was treated with diuretics and packed-red-cell transfusion and was discharged. No diagnosis was reached at this time, and it was appreciated that, unless there was an unexpected maintained improvement, the investigations, especially of the bone marrow, would have to be repeated. He was re-admitted one month later feeling very unwell and with generalized lymphadenopathy. The liver was, however, only palpable to 1 cm below the costal margin.

However, when the bone marrow aspiration was repeated, it showed a heavy infiltration of lymphoid cells with a basophilic cytoplasm and containing well-defined vacuoles, both cytoplasmic and nuclear. The nuclei contained readily identifiable nucleoli. Many of the cells showed block positivity on staining with periodic acid–Schiff (PAS).

Lymph-node biopsy from the neck showed replacement by sheets of what were described as mature-looking lymphocytes with scanty cytoplasm. Moderate numbers of mitoses were seen. The appearances were those of a moderately well-differentiated lymphocytic lymphoma. Despite the doubts as to the exact nature of the patient's lymphoma/leukaemia, he was treated for the next 5 months with intensive chemotherapy, which induced remission.

After 5 months of remission he began to feel generally unwell and complained of bone pain.

A blood smear again showed no abnormal cells, but a bone marrow aspirate showed 80% lymphoblasts. It was from this sample that the marrow culture was initiated.

Remission was induced again, but lasted only 3 months. Further attempts at remission induction failed, and the patient died 3 weeks later.

*Tissue culture.*—The culture (Line 190) was initiated from the bone marrow aspirate obtained during the child's first relapse. Separation of the leucocytes was carried out using a Ficoll-gradient technique (Berrebi *et al.*, 1972). RPMI-1640 medium, supplemented with 10% foetal bovine serum and antibiotics, was used as growth medium. The cells were grown in stationary cultures in loosened screw-topped 125-ml Erlenmeyer flasks. They were incubated at 37 °C, in 5% $CO_2$ in air. The medium was changed every fifth day by aspiration from the upper half of the culture fluid, followed by replacement with fresh growth medium. The studies outlined in this paper were carried out 8 months after the cells were placed in culture.

*Cytological and cytochemical studies.*—Coverslip smears, or cytocentrifuged deposits prepared from the cultured cells, were stained with May–Grünwald Giemsa (MGG) and Leishman for morphological examination. In addition, cytochemistry for the following reactions was carried out, using standard methods (Hayhoe and Cawley, 1972): Sudan black, periodic acid–Schiff (PAS) and reactions for acid phosphatase. For ultrastructural examination the cultured cells were prepared according to published procedures (Cawley and Hayhoe, 1973).

*Karyotype analysis.*—Analysis of the chromosomes after 8 months' growth *in vitro* was carried out as described previously (Karpas *et al.*, 1971).

*Immunological properties and tests for EBNA.*—Fresh cells from the patient, as well as those that had been cultured, were tested for surface thymus-derived (T) lymphocyte receptors by rosette formation with sheep red blood cells (E). Similarly, cultured cells were tested for rosette formation with mouse red blood cells. Immunoglobulin Fc-receptor was tested for rosette formation with IgG-coated ox red blood cells (EA). Tests for receptor sites for the third component of complement (C3) were performed by rosette formation with C3-coated ox red blood cells (EAC). Fluorescein-conjugated polyvalent antisera to immunoglobulin (heavy and light chains) were used to determine the presence or absence of SmIg. The technical details are similar to those described earlier (Gordon *et al.*, 1977).

In addition, the cells were incubated with antiserum to a glycoprotein antigen complex of 23,000 and 30,000 dalton subunits (p23,30) (Schlossman *et al.*, 1976).

The test for the presence of Epstein–Barr viral nuclear antigen was performed according to the method of Reedman and Klein (1973) using known EBNA-positive (B-cell) and EBNA-negative (T-cell) lines as controls.

*Biosynthetic studies.*—$2 \times 10^6$ cultured cells were washed with lysine-free medium and suspended in 2 ml of lysine-free RPMI-1640 medium. This medium was supplemented with 10% dialysed foetal bovine serum and 10 $\mu$Ci of L-[Cl-$^{14}$C]-lysine monohydrochloride (Radiochemical Centre, Amersham, U.K.). Incubation at 37 °C in 5% $CO_2$ in air was continued for 20 h. Cells were spun down and then separated by slow-speed centrifugation. The supernatant was then spun at 10,000 rev/min for 20 min to remove cell debris. This supernatant was then layered in 50-$\mu$l quantities on SDS-acrylamide gel for electrophoresis (Laemmli, 1970). $^{14}$C-labelled human IgMK, produced by a human lymphoid cell line, was used as control.

### RESULTS

### *Cytology and cytochemistry*

Microscopic examination of MGG-stained cytocentrifuged cells revealed a population of small lymphoblasts with a high nuclear/cytoplasmic ratio. The majority of the cells were mononuclear, with an occasional binucleated cell. There was strong block positivity for PAS (Fig. 2). (All figures are of cells cultured *in vitro* for 8 months). The cytoplasm contained numerous azurophilic granules and many cells were vacuolated. Microscopically, the most striking feature was the pleomorphism of the nuclei. They were either round or densely cloven (Fig. 1). The chromatin pattern ranged from an open light appearance to clumped and compact. Occasionally, cytoplasmic fragments could be seen. These were

FIG. 1.—MGG-stained cells showing mononuclear cells, two with cleaved nuclei. ×1300.

FIG. 2.—PAS-stained cells showing block positivity. A binucleated cell can be seen. ×1300.

FIG. 3.—Ultrastructure of a typical lymphoblast with a high nucleus/cytoplasm ratio. Numerous electron-dense glycogen granules can be seen in the cytoplasm (▲). ×14,000.

made up of a membrane-bound cytoplasmic matrix containing material which stained red in MGG.

Ultrastructural examination of the cells revealed what appeared to be typical lymphoblasts, indistinguishable from those found in fresh null-cell ALL, with abundance of glycogen (Fig. 3). Many cells contained in their cytoplasm spherical bodies with an amorphous electron-dense cortex and an almost transparent core (Fig. 4). These resemble the lipid bodies described by Achong and Epstein (1966). In addition, the cytoplasm of some cells contained membrane-bound dense granules (Fig. 4) which could be easily distinguished from mitochondria. Under the electron microscope the minicells did not reveal any unusual features in addition to those seen by light microscopy (Fig. 5).

Microtubules could be seen in the cytoplasm, near the nuclear membrane (Fig. 6) as has been described earlier in Rieder cell formation of ALL (Cawley and Hayhoe, 1973). Occasionally, cells with a thread of nuclear membrane, connecting two nuclei, could be seen (Fig. 7).

In two of about 50 cells examined in detail, a cytoplasmic vesicle containing electron-dense particles of equal size could be seen (Fig. 4).

*Immunological properties and test for EBNA*

Neither the patient's lymphoblasts nor the cells grown *in vitro* formed rosettes

FIG. 4.—Ultrastructure of a mononuclear cell. The cytoplasm contains vacuolated lipid bodies (L), membrane-bound dense granules (G), mitochondria (M), a vesicle containing numerous electron-dense ring-shaped particles ( ↑ ) (and inset). A nuclear bleb can be seen (n). × 16,000.

FIG. 5.—Ultrastructure of the minicells, showing that they are made up of cytoplasmic matrix, some containing electron-dense material which, in one minicell (↑) appears to be clearly membrane-bound. ×19,500.

in any of the tests; nor did they show surface fluorescence with anti-Ig sera. 8% of cultured cells formed rosettes with mouse red blood cells. The nuclei failed to fluoresce in the EBNA test. The cultured cells reacted with the P23, 30 antiserum, whilst the cultured T-cells (Line 45) failed to do so.

*Biosynthetic studies*

The culture medium containing [$^{14}$C] lysine did not reveal any radioactive bands in the SDS-polyacrylamide gel, indicating that the null-cell line does not secrete immunoglobulin. In contrast, the medium harvested from a 24-h growth of the IgMk-synthesizing B-cells formed two bands each for heavy and light chains.

*Karyotype analysis*

The karyotype of the patient's cells was not examined. However, when spreads were prepared from cells grown *in vitro* for 8 months, abnormal karyotypes could be detected. Of the 10 good spreads counted, 6 contained 47 chromosomes, two 48 and two 46 chromosomes. Occasionally a fragmented spread could be seen.

DISCUSSION

The malignant cell population of most patients with acute lymphoblastic leu-

FIG. 6.—Ultrastructure of a cleaved mononuclear cell showing large nuclear blebs (n), lipid bodies (L) and cytoplasmic microtubules (↑) near the nuclear membrane (and inset, ▲). ×11,000.

kaemia (ALL) is made up of EBNA-negative lymphoid cells which have no detectable receptors characteristic of either B- or T-cells, and are therefore classified as null cells.

T-cell ALL is rare in adult patients but approximately 20% of childhood ALL is made up of T cells. We have established a continuous culture of leukaemic T cells from the bone marrow of a child with T-cell ALL (Karpas *et al.*, 1977).

On the other hand, most of the lymphoblastoid cell lines which we and others have established from "null-cell" ALL are EBNA-positive and have SmIg and are therefore classified as B-cells. The cultures of EBNA-positive B-cell lymphoblasts contain large undifferentiated blast cells with a low nuclear/cytoplasmic ratio. They are thought to represent an outgrowth of the patients EBV-infected B cells. On the other hand, they might represent a morphological and biological transformation of normal null cells following their infection with EBV. This might be possible *in vitro* in the absence of circulating antibodies to EBV.

Therefore, the EBNA-negative continuous-cultured null cells reported here should help to determine whether null cells can be infected by EBV, and if so, what kind of transformation occurs as a result of EBV infection *in vitro*.

Our null-cell line was established from the bone marrow of a child with an apparent null-cell lymphoma/leukaemia. However, since the patient was very ill for at least 4 months before his bone marrow involvement became evident, his malignant cells might not have been bone marrow-derived null cells.

FIG. 7.—Ultrastructure of a binuclear cell, showing a thread of nuclear membrane connecting both nuclei. Large aggregates of glycogen can be seen in the cytoplasm (▲). ×14,000.

The cells' morphological, cytochemical and immunological properties after 8 months *in vitro* are very similar to those of the patient's malignant cell population. The Table summarizes those properties and compares them to the properties of two other haemic cell lines which are thought to represent an outgrowth of the leukaemic cells.

The presence of p23,30 antigens on the surface of the cultured cells confirms that the cells are not thymus-derived. Furthermore, since 15–20% of null-cell ALL contain these antigens (Schlossman *et al.*, 1976) its presence does not negate the null-cell classification of our culture.

The leukaemic cells were not analysed for their karyotype at diagnosis, but the cultured cells revealed abnormal chromosomal spreads with many karyotypes containing 47 chromosomes.

A study of karyotypes in acute leukaemia (Sandberg *et al.*, 1968) revealed that the most frequent abnormal karyotypes in ALL had 47 chromosomes, with many cases of aneuploidy. Since most of our B-cell lines and one T-cell line from leukaemic patients (Karpas *et al.*, 1977) have a normal karyotype, the presence of these abnormal chromosomes in our null-cell cultures lends further support to the assumption that the null-cell line is a malignant one.

Additional support for this assumption is provided by the ultrastructural studies. Nuclear blebs which have been found in many of the cells have been described as a common factor in malignant haemic

TABLE .—*The Properties of Unusual Leukaemia-derived Haemic Cell Lines*

| Cell line | Source of culture* | Original disorder | Surface markers (%) SIg | E | Fc | C3 | EBNA | Cytochemistry % PAS | Acid phosphatase | References |
|---|---|---|---|---|---|---|---|---|---|---|
| 190 | BM | Null-cell ALL | — | — | — | — | — | 100 | — | |
| 45 | BM | T-cell ALL | — | 2–10 | — | — | — | 10 | + | Karpas *et al.*, 1977 |
| K562 | PE | Blastic crisis of CML | — | 5–9 | 90–95 | 3–9 | — | — | + | Lozzio & Lozzio 1975<br>Klein *et al.*, 1976 |

*BM—bone marrow: PE—Pleural effusion.

cells (Achong and Epstein, 1966; Anderson, 1966; Dorfman, 1967; McDuffie, 1967). A recent study by Ahearn *et al.* (1974) shows that nuclear blebs are associated with aneuploidy in human acute leukaemia, and that they cannot be found in normal bone marrow. Likewise, the appearance of Rieder-cell formation has been reported to be a feature of leukaemic cells rather than normal cells (Cawley and Hayhoe, 1973). In addition the esterase cytochemistry, kindly performed by Dr Higgy, showed a null-cell pattern of positivity (Higgy, Burns and Hayhoe, 1977).

The cytoplasmic vesicles which contained electron-dense particles resembled those we found in rat cells which carry the avian and murine sarcoma viral genomes (Karpas, Cawley and Tuckerman, 1972). They might therefore represent a morphological expression of latent incomplete oncorna viral information. A continuous prolonged culture or co-cultivation with permissive cells might result in the expression of, and productive replication of, a genuine human oncorna virus.

The clinical picture at presentation and its subsequent course are not unlike those of leucosarcomatosis. This unique culture could provide information as to the origin of the malignant haemic null cells.

We are grateful to Professor F. G. J. Hayhoe for his continuous interest, to Dr N. Barnes for providing the clinical material, to Mr R. J. Flemans for help with the cytochemical staining and to Mr J. P. Emmines for the electron micrographs. We thank Dr T. Springer for the anti-p23, 30 serum.

This work was supported by the Leukaemia Research Fund (U.K.).

### REFERENCES

ACHONG, B. G. & EPSTEIN, M. A. (1966) Fine Structure of the Burkitt Tumor. *J. natn. Cancer Inst.* **36**, 877.

AHEARN, M. J., TRUJILLO, J. M., CORK, A., FOWLER, A. & HART, J. S. (1974) The Association of Nuclear Blebs with Aneuploidy in Human Acute Leukaemia. *Cancer Res.*, **34**, 2887.

ANDERSON, D. R. (1966) Ultrastructure of Normal and Leukemic Leukocytes in Human Peripheral Blood. *J. Ultrastruct. Res.*, **9**, 24.

BERREBI, A., OBERLING, F., TONGIO, N. M. & MAYER, S. (1972) Isolement des Cellules Blastiques de la Moelle Osseuse Leucémique par Centrifugation en Gradient de Densité Isopaque-Ficoll. *Path. Biol.*, **20**, 727.

CAWLEY, J. C. & HAYHOE, F. G. J. (1973) *Ultrastructure of Haemic Cells.* Philadelphia: W. B. Saunders Co.

DORFMAN, R. F. (1967) The Fine Structure of a Malignant Lymphoma in a Child from St. Louis, Missouri. *J. natn. Cancer Inst.*, **38**, 491.

GORDON, J., HOUGH, D., KARPAS, A. & SMITH, J. L. (1977) Immunoglobulin Expression and Synthesis by Human Haemic Cell Lines. *Immunology*, **32**, 559.

HAYHOE, F. G. J. & CAWLEY, J. C. (1972) Acute Leukaemia: Cellular Morphology, Cytochemistry and Fine Structure. *Clinics in Haemat.*, **1**, 49.

HIGGY, K. E., BURNS, G. F. & HAYHOE, F. G. J. (1977) Discrimination of B, T and Null Cell Lymphocytes by Esterase Cytochemistry. *Scand. J. Haemat.*, **18**, 437.

KAPLAN, J., SHOPE, T. C. & PETERSON, W. L. (1974) Epstein–Barr Virus-negative Human Malignant T-cell Lines. *J. exp. Med.*, **139**, 1070.

KARPAS, A., CAWLEY, J. C., TUCKERMAN, E., FLEMANS, R. J. & HAYHOE, F. G. J. (1971) Cytochemistry, Cytogenetics and Ultrastructure of Hamster Tumour Cells Carrying Mouse Sarcoma Viral Genome (HT-1 Cells). *Br. J. Cancer*, **25**, 779.

KARPAS, A., CAWLEY, J. & TUCKERMAN, E. (1972) Cytological, Cytogenetic and Ultrastructural Studies of Rat Cell Lines Carrying Avian and

Murine Sarcoma Genomes. *Z. Krebsforsch.*, **78**, 51.

KARPAS, A., HAYHOE, F. G. J., GREENBERGER, J. S., BARKER, C. R., CAWLEY, J. C., LOWENTHAL, R. M. & MOLONEY, W. C. (1977) The Establishment and Cytological, Cytochemical and Immunological Characterisation of Human Haemic Cell Lines: Evidence for Heterogeneity. *Leukemia Res.*, **1**, 35.,

KLEIN, G., LINDAHL, T., JONDAL, M., LEIBALD, W., MENEZES, J., NILSSON, K. & SUNDSTROM, C. (1974) Continouus Lymphoid Cell Line with Characteristics of B Cells (Bone Marrow Derived) Lacking the Epstein–Barr Virus Genome Derived from Three Human Lymphomas. *Proc. natn. Acad. Sci., U.S.A.*, **71**, 3283.

KLEIN, E., BEN-BASSAT, H., RALPH, P., ZEUTHEN, J., POLLIACK, A. & VANKY, F. (1976) Properties of the K562 Cell Line, Derived from a Patient with Chronic Myeloid Leukaemia. *Int. J. Cancer*, **18**, 421.

LAEMMLI, U. K. (1970) Cleavage of Structural Proteins during the Assembly of the Head of Bacteriophage T4. *Nature, Lond.*, **227**, 680.

LOZZIO, C. B. & LOZZIO, B. B. (1975) Human Chronic Myelogenous Leukaemic Cell-line with Positive Philadelphia Chromosome. *Blood*, **45**, 321.

MCDUFFIE, N. G. (1967) Nuclear Blebs in Human Leukaemic Cells. *Nature, Lond.*, **214**, 1341.

MINOWADA, J., OHNUMA, T. & MOORE, G. E. (1972) Rosette-forming Human Lymphoid Cell Lines. 1. Establishment and Evidence for Origin of Thymus-derived Lymphocytes. *J. natn. Cancer Inst.*, **49**, 891.

REEDMAN, B. M. & KLEIN, G. (1973) Cellular Localization of an Epstein–Barr Virus (EBV)-associated Complement-fixing Antigen in Producer and Non-producer Lymphoblastoid cell Lines. *Int. J. Cancer*, **11**, 499.

SANDBERG, A. .A, TAKAGI, N., SOFUNI, T. & CROSSWHITE, L. H. (1968) Chromosomes and Causation of Human Cancer and Leukaemia. V. Karyotypic Aspects of Acute Leukaemia. *Cancer, N.Y.*, **22**, 1268.

SCHLOSSMAN, S. F., CHESS, L., HUMPHREYS, R. E. & STROMINGER, J. L. (1976) Distribution of Ia-like Molecules on the Surface of Normal and Leukemic Human Cells. *Proc. natn. Acad. Sci. USA*, **73**, 1288.